# Natural agents inhibit colon cancer cell proliferation and alter microbial diversity in mice

**DOI:** 10.1371/journal.pone.0229823

**Published:** 2020-03-20

**Authors:** Lulu Farhana, Sarah Sarkar, Pratima Nangia-Makker, Yingjie Yu, Pramod Khosla, Edi Levi, Asfar Azmi, Adhip P. N. Majumdar

**Affiliations:** 1 John D Dingell Veterans Affairs Medical Center, Detroit, Michigan, United States of America; 2 Department of Internal Medicine, Wayne State University School of Medicine, Detroit, Michigan, United States of America; 3 Karmanos Cancer Institute, Detroit, Michigan, United States of America; 4 Department of Nutrition and Food Science, Wayne State University, Detroit, Michigan, United States of America; 5 Department of Pathology, Wayne State University School of Medicine, Detroit, Michigan, United States of America; Beckman Research Institute, UNITED STATES

## Abstract

The current study was undertaken to investigate the effect of differentially formulated polyphenolic compound Essential Turmeric Oil-Curcumin (ETO-Cur), and Tocotrienol-rich fraction (TRF) of vitamin E isomers on colorectal cancer (CRC) cells that produce aggressive tumors. Combinations of ETO-Cur and TRF were used to determine the combinatorial effects of ETO-Cur and TRF-mediated inhibition of growth of CRC cells *in vitro* and HCT-116 cells xenograft in SCID mice. 16S rRNA gene sequence profiling was performed to determine the outcome of gut microbial communities in mice feces between control and ETO-Cur-TRF groups. Bacterial identifications were validated by performing SYBR-based Real Time (RT) PCR. For metagenomics analysis to characterize the microbial communities, multiple software/tools were used, including Quantitative Insights into Microbial Ecology (QIIME) processing tool. We found ETO-Cur and TRF to synergize and that the combination of ETO-Cur-TRF significantly inhibited growth of HCT-116 xenografts in SCID mice. This was associated with a marked alteration in microbial communities and increased microbial OTU (operation taxonomic unit) number. The relative abundance of taxa was increased and the level of microbial diversity after 34 days of combinatorial treatment was found to be 44% higher over the control. Shifting of microbial family composition was observed in ETO-Cur-TRF treated mice as evidenced by marked reductions in *Bacteroidaceae*, *Ruminococcaceae*, *Clostridiales*, *Firmicutes and Parabacteroids* families, compared to controls. Interestingly, during the inhibition of tumor growth in ETO-Cur treated mice, probiotic *Lactobacillaceae* and *Bifidobacteriaceae* were increased by 20-fold and 6-fold, respectively. The relative abundance of anti-inflammatory *Clostridium XIVa* was also increased in ETO-Cur-TRF treated mice when compared with the control. Our data suggest that ETO-Cur-TRF show synergistic effects in inhibiting colorectal cancer cell proliferation *in vitro* and in mouse xenografts *in vivo*, and might induce changes in microbial diversity in mice.

## Introduction

Despite recent therapeutic advances, colorectal cancer (CRC) remains the third most common cancer in the US with nearly 50% of patients eventually developing recurrent tumors, which are resistant to routine chemotherapy. This underscores a need to develop novel strategies to combat chemo-resistance in cancer cells, which produce highly aggressive colon tumors.[1] The overall 5-year survival rate of CRC is approx. 60% and recurrent malignancy after initial treatment is 30–40% in patients with CRC.[2–4] Most recurrences happen during the first 2 to 3 years after initial treatment.[3, 5]

Curcumin, a lipophilic polyphenolic compound, with a broad medicinal value, is the major active ingredient of *Curcuma Longa* (turmeric).[6] Curcumin's pharmacological effects have been documented in various diseases including gastrointestinal and neurological disorders, as well as diabetes, hepatic, cardiovascular, Alzheimer’s disease, pancreatitis, cystic fibrosis, inflammatory bowel disease, arthritis, multiple sclerosis, and many types of cancer.[6–11] While numerous *in vitro* and animal models of colon cancer have found Curcumin to be a chemo-preventive and chemo-therapeutic agent, clinical trials have failed to duplicate Curcumin’s anti-cancer property.[11] Although Curcumin- induced sensitization of cancer cells to chemotherapy has been reported, there are major concerns regarding bioavailability of Curcumin based on *in vivo* studies.[12]

Due to its poor bioavailability;[12] efforts are underway to produce different analogs or formulations of Curcumin with increased bioavailability. ETO-Curcumin (ETO-Cur) is one such formulation, where Curcumin is complexed with essential turmeric oil (Dolcas Biotech; Chester, NJ*)*. ETO-Cur has been shown to be 7–8 times more bioavailable with superior anti-cancer properties than standard Curcumin.[13–15]

The Vitamin E family consists of eight distinct isomers– 4 tocopherols and 4 tocotrienols [16] Tocotrienols display potent antioxidant, anticancer, anti-inflammatory, neuroprotective and cholesterol-lowering properties.[17–23] A tocotrienol-rich fraction (TRF), (containing a mixture of isomers) isolated from palm oil has been used as an anti-oxidant and anti-cancer agent.[24–27]

Given the sheer vastness of microflora in the gut and numerous arrays of species and metabolites produced, it is likely that bacteria plays a pivotal role in many gastrointestinal diseases including CRC.[28–33] Human colon harbors a complex microbial flora containing approximately 10^14^ bacteria consisting of 10^3^ different microbial species that maintain homeostasis.[34]

The primary objectives of the current pre-clinical investigation were to determine whether synergistic ETO-Cur-TRF inhibits the growth of colorectal cancer cells in vitro and SCID mice xenograft and to elucidate the underlying regulatory process by focusing on the ETO-Cur-TRF mediated role of gut microbiome.

## Methods

### Cell lines and reagents

Human colon cancer cells HT-29 and HCT-116 were obtained from the American Type Culture Collection (ATCC, Rockville, MD). The cells were maintained in Dulbecco’s modified Eagle’s medium (DMEM) supplemented with 10% fetal bovine serum (FBS) (Invitrogen, Grand Island, NY) and 1% antibiotic/antimycotic in a humidified incubator at 37°C in an atmosphere of 95% air and 5% carbon dioxide. 5-Fluorouracil + Oxaliplatin (FuOx) resistant cells were generated in our laboratory as described earlier [35, 36]. These cells were maintained in DMEM supplemented with FBS containing 2 x FuOx (50μM 5 FU + 1.25 μM Ox) culture medium. The medium was changed twice a week, and cells were passaged using 0.05% trypsin/EDTA (Invitrogen).

ETO-Cur was obtained from Dolcas Biotech, Chester,NJ; (BCM-95, Lot # 1511–39). This product, in addition to Curcumin contained other constituents of turmeric, primarily essential turmeric oils (ETO) comprising of aromatic-tumerones (ar-tumerones), α-turmerones, β-turmerones, α-santalene and aromatic curcumene. TRF composed of 190 mg total tocotrienol (72 mg α-tocotrienol, 10 mg β-tocotrienol; 94 mg δ-tocotienol) + 57 mg α-tocopherol and 0.43 mg mixed carotenes was a product of Carotino, Sdn. Bhd., Malaysia. The capsules were manufactured by Kamata Co. Ltd., Tokyo, Japan (Lot # 161983170105).

### Determination of cell growth

The growth of colon cancer cells was determined by mitochondrial-dependent reduction of 3-(4,5-dimethylthiazol-2yl)-2, 5-diphenyltetrazolium bromide (MTT) (Sigma) to formazan as described previously.[37] Briefly, the cells (5x10^3^) were seeded in quadruplicates onto 24 well culture dishes. After 24 hours, fresh medium containing various concentrations of ETO-Cur and TRF was added. After 72 h, cell survival was determined by MTT assay. The medium was removed from the dishes and the cells were incubated at 37°C with MTT (0.5 mg/mL in 1XPBS) for 4 hours. The medium was then aspirated and the cells were solubilized in 0.04 N HCL in isopropanol. The optical density (OD) was measured at both 570 and 630 nm.

### Analysis of interaction between ETO-Curcumin and TRF

Combination indices (CI) method adapted for *in vitro* drug testing was employed to determine the nature of interaction between ETO-Cur and TRF. The method utilizes multiple drug effect equations which were originally derived from enzyme kinetic methods. The output is represented as CI and/or isobologram analysis. CI analysis was performed by using Calcusyn software (Biosoft, Ferguson, MO). Based on the CI values attained, the extent of synergism/antagonism is determined. Generally, CI values below 1 suggest synergy, whereas CI values above 1 indicate antagonism between the 2 drugs. CI values in the range of 0.9–1.1 would mostly indicate additive effects, values between 0.85–0.9 suggest slight synergy, and values between 0.3–0.7 are indicative of moderate synergy.[38] All values less than 0.3 would suggest strong synergy between the two drugs.[38]

### Tumor growth in SCID mice

All animal experiments were performed according to Wayne State University’s Institutional Animal Care and Use Committee (IACUC) approved protocol #A02-02-13. Animal Welfare Assurance #A3310-01.

Tumors were generated in 4-week-old female SCID mice (Taconic Laboratory) by subcutaneous injections of 1 x 10^6^ HCT-116 cells suspended in 100 μL Matrigel in the flank region on either side. To study the chemo-preventive efficacy of ETO-Cur and TRF combination, animals were given 5 mg/kg ETO-Cur and 2 mg/kG TRF in 100 μL sesame oil by oral gavage 7 days after inoculation of cells. ETO-Cur-TRF treatment, given 5 days a week (Monday to Friday) was continued until the animals were sacrificed. The animals in the control group were given only sesame oil by oral gavage. Tumor volumes were calculated as previously described [37]. Feces were collected at the start and during the experimental period to analyze changes in gut microbiome. Mice were monitored regularly for any signs of discomfort. At the end of the treatment period, all animals were sacrificed by CO_2_ inhalation. Assurance of death was performed by cervical dislocation.

### DNA extraction for 16S rRNA microbiota community profiling

Genomic DNA was extracted from mice feces using QIAamp DNA Stool Mini Kit (Qiagen, CA, USA) according to the manufacturer’s instruction and as described.[39] Mice feces in ASL buffer were thoroughly vortexed and the suspension was heated for 15 min at 70 ^0^C. DNA was isolated from this suspension according to manufacturer’s protocol. Purified DNA was used for analysis of 16S rRNA community profiling, which was performed by LC Sciences (Houston, Texas, USA) on fee per basis. The conserved regions of V3 and V4 of the 16S rRNA gene were amplified using PCR. After one cycle of PCR, sequencing adapters and barcodes were added for further amplification of the library for sequencing. Bacterial 16S rDNA universal primer (338F: 5’-ACTCCTACGGGAGGCAGCAG-3’; 806R: 5’-GGACTACHVGGGTWTCTAAT-3’) was used to generate amplicons of approximately 469 bp using Phusion Hot start Master Mix (New England Biolab, USA). PCR condition for 16S fragment amplification was consisted of initial denatuation 98°C for 30sec, followed by 35 cycles of 98°C for 10sec, 64°C for 30sec, 72°C for 45sec, with a final extension at 72°C for 10 min. PCR amplification products were detected by 2% agarose gel electrophoresis and target fragments were recovered using the AxyPrep PCR Cleanup Kit (Fisher Scientific, USA).

The PCR product was further purified using the Quant-iT PicoGreen dsDNA Assay kit (Therofisher Scientific, USA). The library was quantified using the Promega QuantiFluor Fluoresence quantification system. Each qualified sequencing library (above 2 nM) was diluted and pooled/multiplexed. The pooled library was loaded on Illumina MISeq system and MISeq Reagent Kit V3 (600 cycles) was used for pair-end sequencing (2x300 cycles). Raw reads were obtained from MiSeq in the FASTQ format and the Phred score was in the Sanger format (Illumina CASAVA version 1.8) was used for base calling. For raw data processing, the data analysis software FLASH (Fast Length Adjustment of Short reads) v1.2.8 was used to merge the pair-end reads and Vsearch was used to remove chimera sequences. The filtered sequences were clustered into operational taxonomic units (OTUs) based on 97% sequence similarity. The Biological Observation Matrix (BIOM) format, QIIME (Quantitative insights into microbial ecology) was used to save the abundance and taxa information for each sample. R (OTU) statistics was used for each sample statistics. For alpha and beta diversity analysis, OTUs abundance was used to assess the diversity of bacteria in each sample. For taxonomic analysis OTUs were mapped to RDP and NT-16S for bacterial taxonomy.

#### Real-Time PCR

For Real Time PCR, DNA was analyzed in triplicate using the 2× Power-Up SYBR Green PCR Master Mix (Applied Biosystems) and the ABI Prism 7500 sequence detection system. PCR consisted of 40 cycles of 95°C for 10 min and then 95°C for 15 sec, 60°C for 60 sec. The primer sequences of *Bifidobacteria* and *Lactobacillus* were used to evaluate the presence of specific types of bacteria [39]. Ct values were normalized to assess the relative concentration of specific DNA for each sample as described by the manufacturer. Each sample ^ΔΔ^CT values were calculated by normalizing to the CT value of total bacteria (Eubacteria) [40]. All primers were synthesized by Integrated DNA Technology Inc. (Coralville, IA, USA).

### Statistical analysis

For microbiota data statistical analysis, Metastats software for metagenomics sequencing data from two groups to characterize the microbial communities was utilized. CD-HT and R statistical software was used for BIOM-formatted OTU communities clustering and OTU statistics. For examining alpha diversity, QIIME software was used for graphics and statistical purposes. RDP classifier, QIIME, TUIT GraPhlAn, MetaPhlAn, R software/Too were used for taxonomic classification and statistics.

Statistical analysis was performed using Microsoft Excel. Unless otherwise stated, data were expressed as mean +/- SD. For Real Time PCR data, standard deviation of mean between two groups and t-test were performed to determine the significance level between two groups. The p-values were calculated using 2 sample t-test, assuming unequal variance. Values <0.05 were considered statistically significant.

## Results

### ETO-Curcumin and TRF show synergistic effect

**Fig 1:**
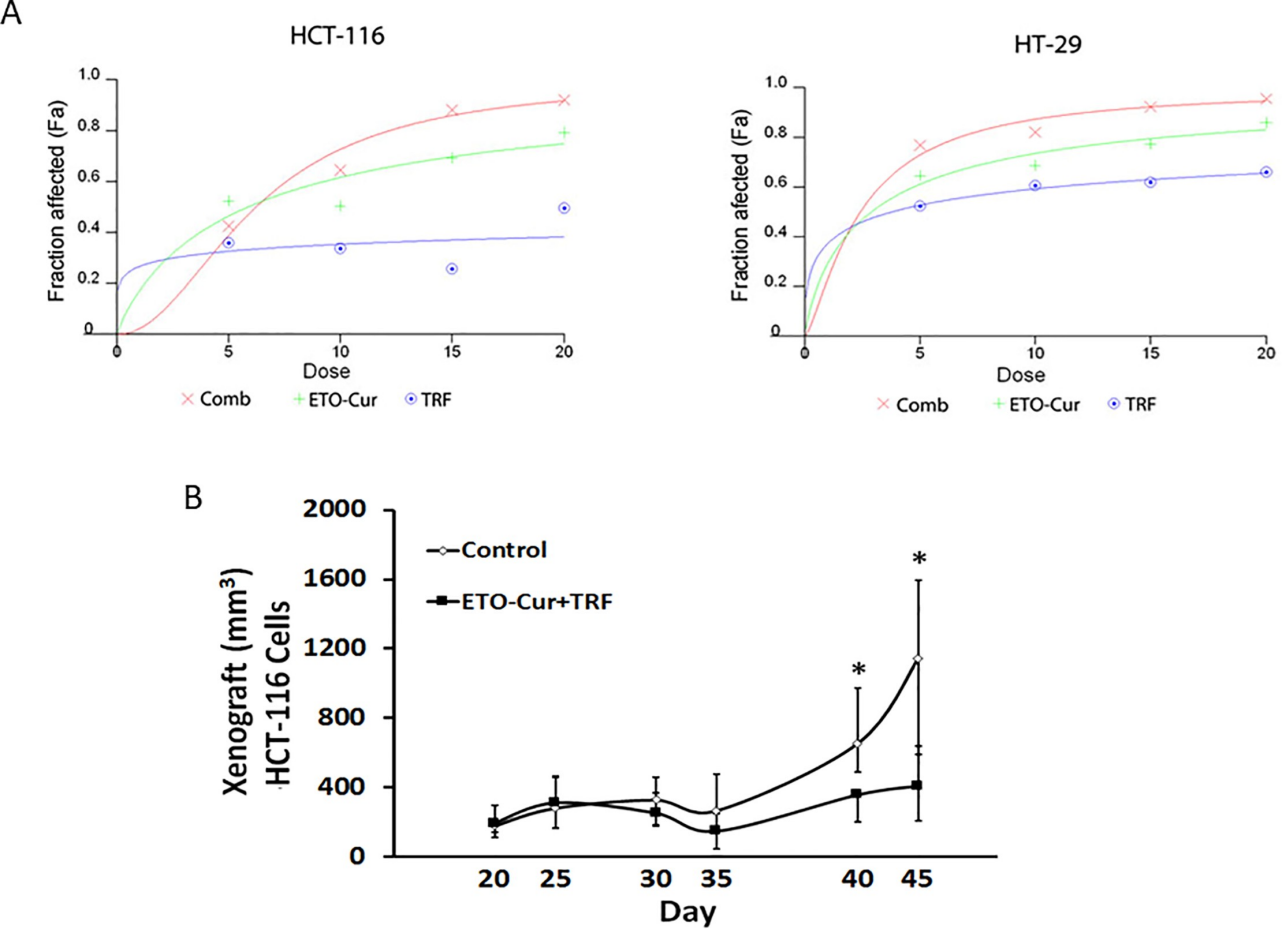
Dose response curves for ETO-Cur and TRF in HCT-116 and HT-29 cells produced by fixed ratio method (A). The cells were treated with increasing doses of ETO- Cur and TRF for 72 h in quadruplicate. MTT assay was performed to calculate viable and the affected cells. Fa represents the fraction of cells affected in response to the treatment. Fa for combination treatment was higher than either agent alone. Fa values were used to calculate synergy using CalcuSyn software as described in Materials and Methods. CI values < 0.9 suggest synergy. Tumor growth in SCID mice injected with HCT-116 cells (B). The mice were given a mixture of 5 mg/kg ETO-Cur and 2 mg/kg TRF five days a week by oral gavage in 100 μl sesame oil for 6 weeks. Control animals were given sesame oil alone. Tumors were measured once a week and tumor volumes were calculated using the formula: Tumor volume = length x width x width/2. Each point represents average volume of 8 tumors ± standard deviation. *p value <0.*05*.

Two human colon cancer cells HT-29 and HCT-116 were used to study synergy effects between ETO-Cur and TRF. Data from *in vitro* studies revealed that although ETO-Cur and TRF, each alone inhibited the growth in both HCT-116 and HT-29 cells, the magnitude of inhibition was much greater when both agents were given together (Fig 1A). The effect of the combinatorial treatment was found to be synergistic in both HT-116 and HT-29 colon cancer cells but only at 5 μM and 10 μM levels as evidenced by CI values of ≤0.8 for both cell lines (Table 1). However, maximal synergism revealing a CI value of 0.42 for HCT-116 cells was observed with the lowest level (5 μM) of ETO-Cur and TRF (Table 1). We also found HT-116 cells to be more sensitive to both ETO-Cur and TRF (Fig 1A).

**Table 1:** Synergy analysis for ETO-Cur and TRF combination therapy in colon cancer cells.

Combination Therapy		Combination Index (CI)	
ETO-Cur (μM)	TRF (μM)	HCT116	HT-29
5	5	0.4260	0.7690
10	10	0.6450	0.8200
15	15	0.8820	0.9230
20	20	0.9200	0.9560

Combination index values <1.0 are increasingly supra-additive, and Values >1.0 are increasing less than additive

In HCT-116 xenograft mouse model, combination of ETO-Cur and TRF significantly inhibited tumor growth compared to the control (Fig 1B). Significant inhibition of tumor growth in response to the combinatorial treatment of ETO-Cur and TRF became evident after 40 days (Fig 1B). The current observation suggests a synergistic effect of ETO-Cur and TRF in inhibiting the growth of CRC cells *in vitro* and in mouse xenograft *in vivo*.

### Microbial 16S rRNA profiling of ETO-Cur-TRF treated mice

**Fig 2:**
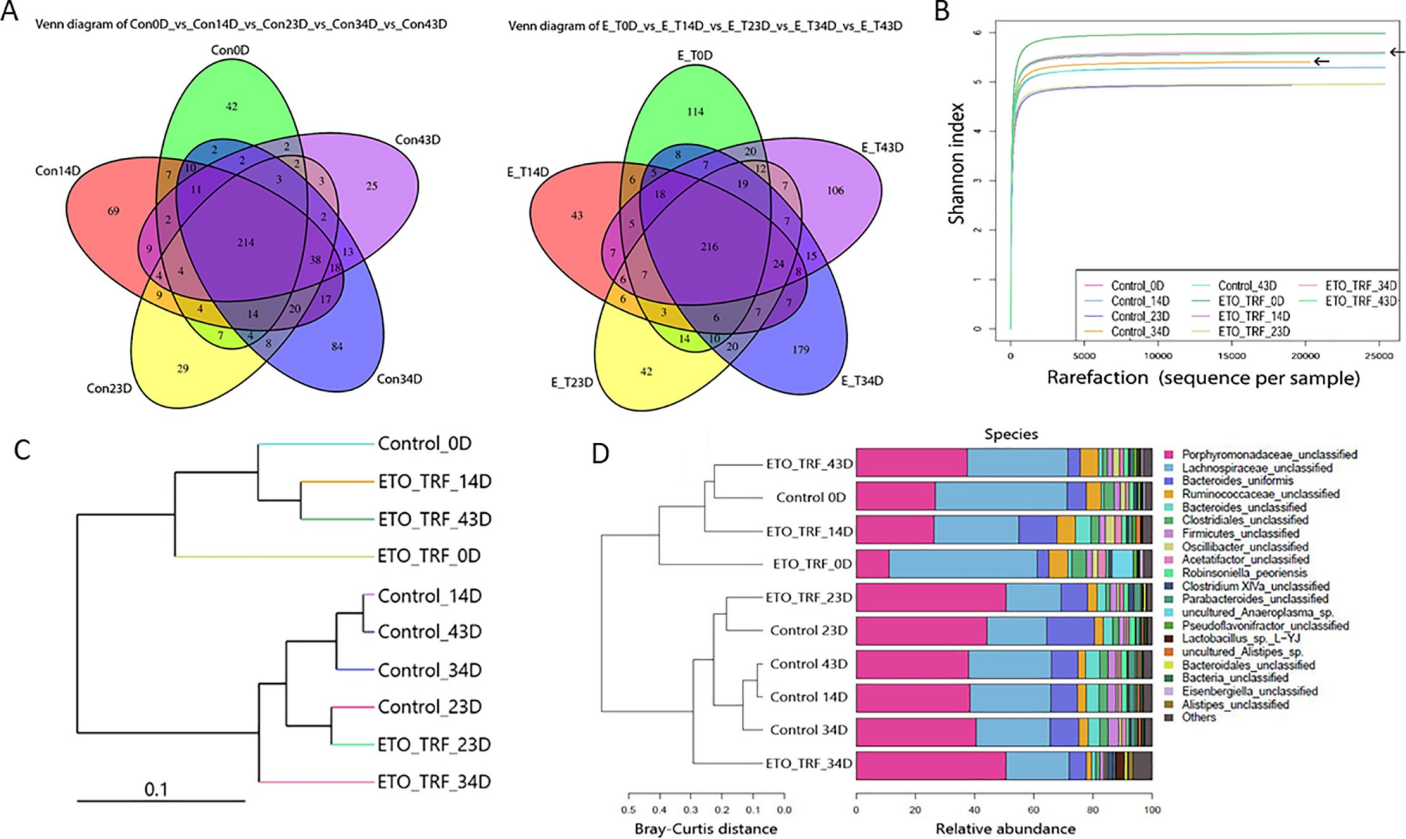
Venn diagram showing the shifting of microbial OTUs between control and ETO-Cur-TRF treated mice feces from 0 days to 43 days (A). Values were obtained from purified DNA pooled from 3–4 mice in each group. Venn diagram shows the similarity and specificity of OTU in different samples/groups. Each circle in Venn diagram stands for a sample. The overlapped region shows the number of common OTUs and non–overlapped region showed the number of common OTUs and non overlapped region shows the number of unique OTUs. Alpha diversity shows that the combination of ETO-Cur-TRF increases species diversity and richness in treated mice. Rarefaction curves are plotted by Shannon index showing the OTUs/species abundance in different days and that the species richness increased on 34day as indicated by arrows (B). Alpha diversity was determined using QIIME. ETO-Cur-TRF treated mice shows the differential distribution of microbial communities. Beta diversity analysis shows the relative abundance of microbial communities using distant matrix as determined by UPGMA Weighted-UniFrac clustering of microbial OTU/species sequence in different samples. The distance suggests differences in microbial composition in samples (C) and Bray-Curtis plot shows the differences of microbial species abundance in community (D).

It is well known that gut microbiome plays a key role in modulating biological activities, bioavailability and bio-efficacy of polyphenols and their metabolites.[41–44] To elucidate changes in microbial communities associated with ETO-Cur-TRF treatment, we sequenced 16S rRNA genes isolated from feces of mice bearing HCT-116 xenograft tumors. Sequencing of the V3+V4 region was used for operation taxonomic unit (OTU) clustering based on 97% sequence similarity. The changes of microbial OTU exhibited during different periods of ETO-Cur-TRF treatment and respective controls revealed that microbial OTU number increased following 34 days of ETO-Cur-TRF treatment (Fig 2A), when tumor inhibition also began to show difference over the control and began to diverge (Fig 1B).

For alpha diversity, Shannon diversity index revealed the distribution of microbial taxa richness. Although the diversity index shows a similar index number between the two groups (Control = 5.40; ETO-Cur-TRF = 5.52), OTU of species revealed a marked increase in the ETO-Cur-TRF group compared to control at 34 day (Fig 2B). Beta diversity in phylogenetic tree showed the relative abundance of microbial composition diverse between ETO-Cur-TRF treated and the control after 34 day of treatment (Fig 2C) and Bray-Curtis plot revealed the dissimilarity of species among microbial communities in samples (Fig 2D).

**Fig 3:**
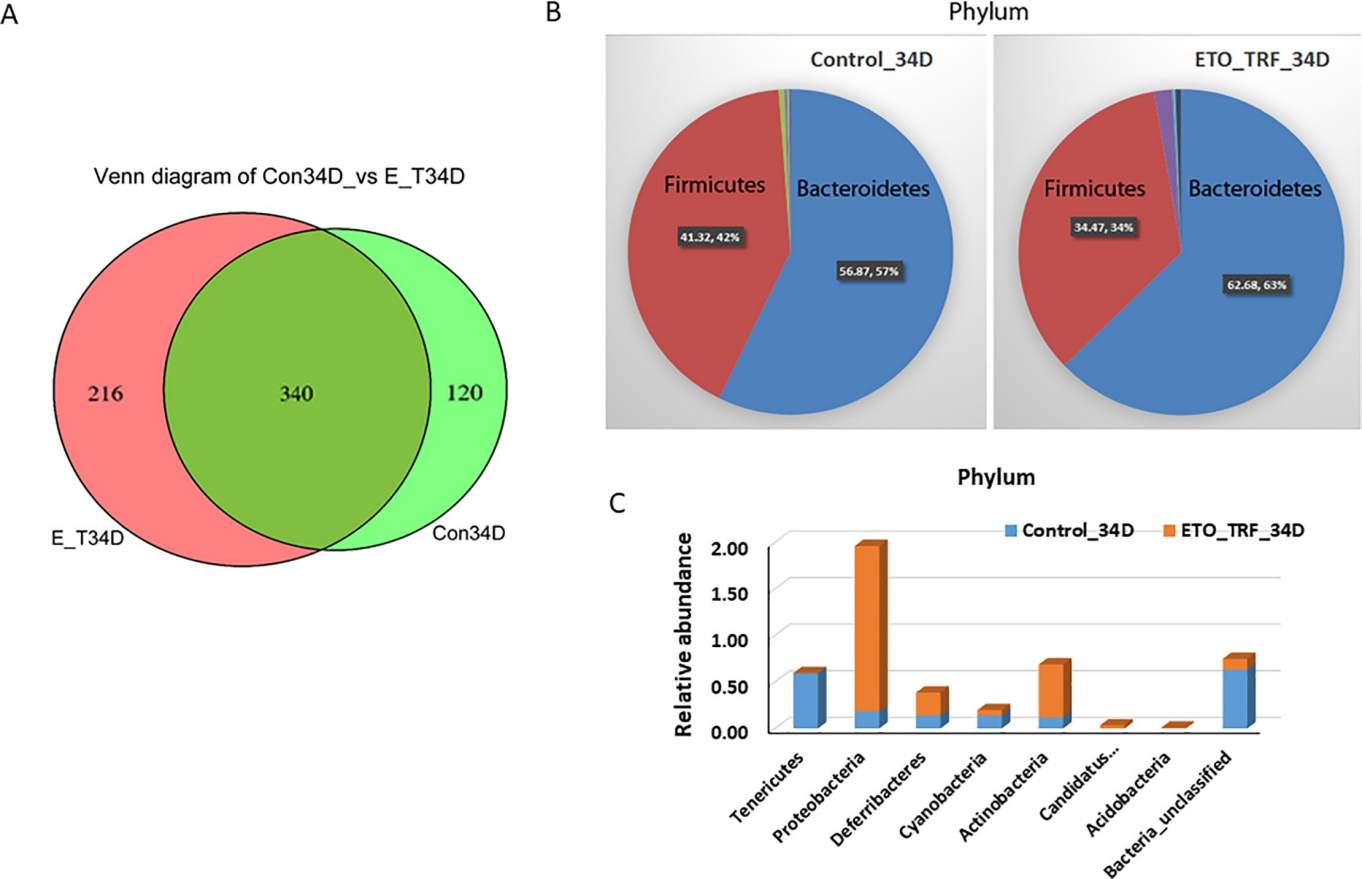
Venn diagram showing shared microbial OTUs between 34 day of ETO-Cur-TRF treated and control mice; ETO-Cur-TRF increased microbial diversity (A) and altered microbial phylum (B and C). Genomic DNA from mouse fecal cells was isolated using QIAamp DNA Stool Mini Kit (Qiagen) according to the manufacturer’s instruction, subsequently used for analysis of 16S rRNA gene community profiling.

Further analysis of the data revealed that an overlap of 340 bacterial species between the control and ETO-Cur-TFR-treated SCID mice and the diversity of species number(n) increased 44% in ETO-Cur-TRF treated mice (216n) compared to control mice (120n) after 34 days (Fig 3A). Thus ETO-Cur-TRF treatment correlated with increased species richness and diversity. Relative abundance of microbial phylum was found to be predominantly *Bacteroides* and *Firmicutes* (97–99%) in both groups of mice (Fig 3B). However, ETO-Cur-TRF significantly increased the abundance of *Proteobacteria* (9.9-fold) and *Actinobacteria* (4.7-fold) (Fig 3C). In the controls, the abundance of *Proteobacteria and Actinobacteria* represented 2% and 0.57% of total microbiota phylum, (Fig 3B). *Tenericute*, *whose* abundance in control represented 0.59%, was totally eliminated in ETO-Cur-TRF treated mice (Fig 3B).

**Fig 4:**
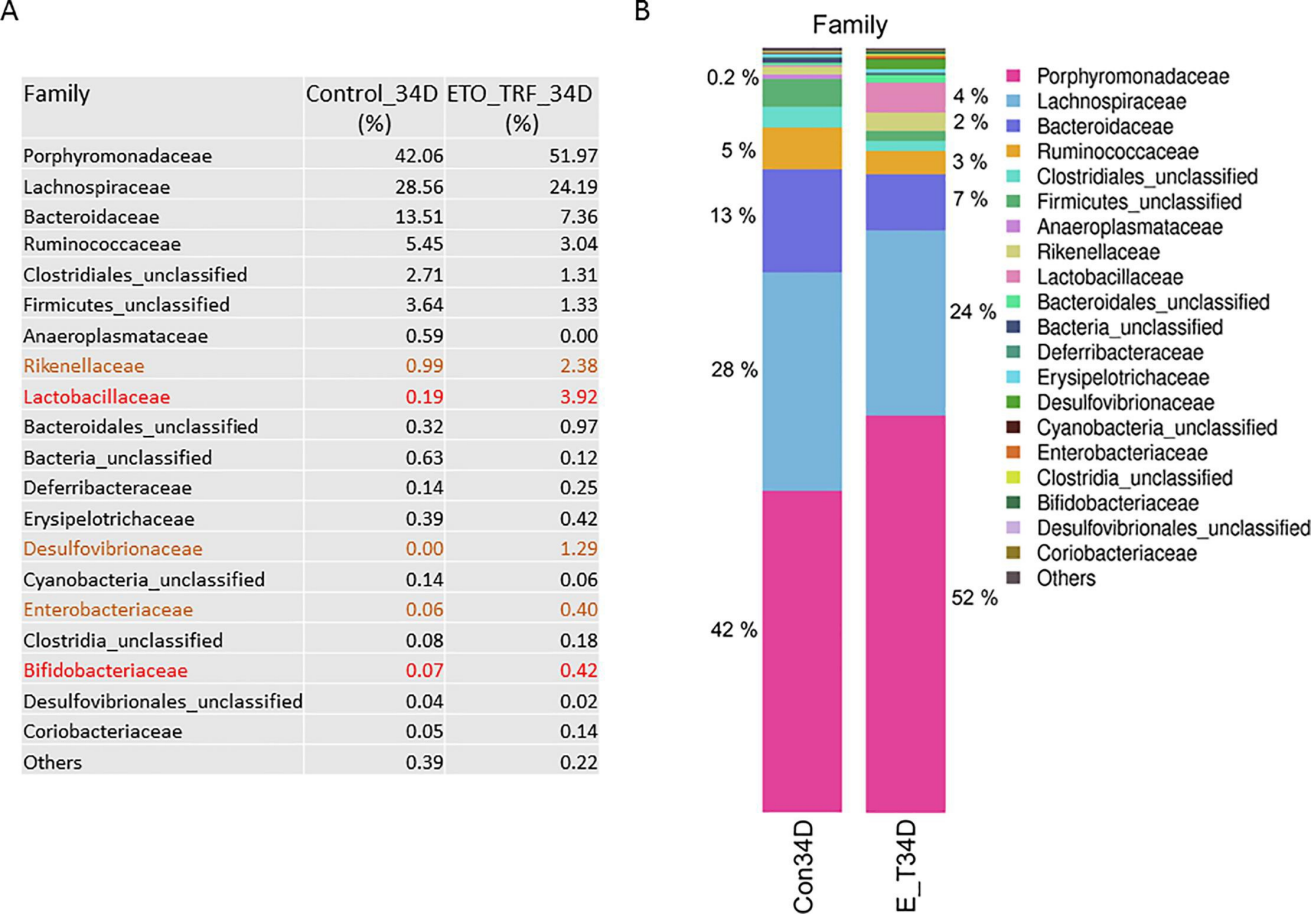
16S rRNA microbial community profiling showing the relative abundance of Family. The changes of microbial family after ETO-Cur-TRF treatment at 34 days when compared with the corresponding control (A). Percent changes in different microbial family between ETO-Cur-TRF treated and the corresponding control (B). Genomic DNA from mouse fecal cells was used to analyze microbial OTU abundance.

The composition of the microbial family was predominantly *Porphydomonadaceae*, *Lachnospiraceae*, *Bacteroidaceae and Ruminococcaceae* in both control and ETO-Cur-TRF treated mice. While 16s RNA gene sequencing revealed that ETO-Cur-TRF increased the abundance of *Porphymonadaceae*, *Rickenellaceae*, *Lactobacillaeceae*, *Desulphovibrionaceae*, *Enterobactericeae and Bifidobacteriaceae* families, it decreased *Bacteroidaceae* significantly that included *Lachnospirace*, *Ruminococcaceae* and *Firmicutes* (Fig 4A and 4B). Microbial diversity decreased by 45% in *Bacteroidaceae* family in ETO-Cur-TRF group after 34 days of treatment, but *Rickenellaceae* increased in the treated group (Fig 4B). Interestingly, probiotic and anti-inflammatory *Lactobacillaceae* and *Bifidobactericeae* was increased 20-fold and 6-fold, respectively in the ETO-Cur-TRF treated mice compared to the corresponding control (Fig 4B).

**Fig 5:**
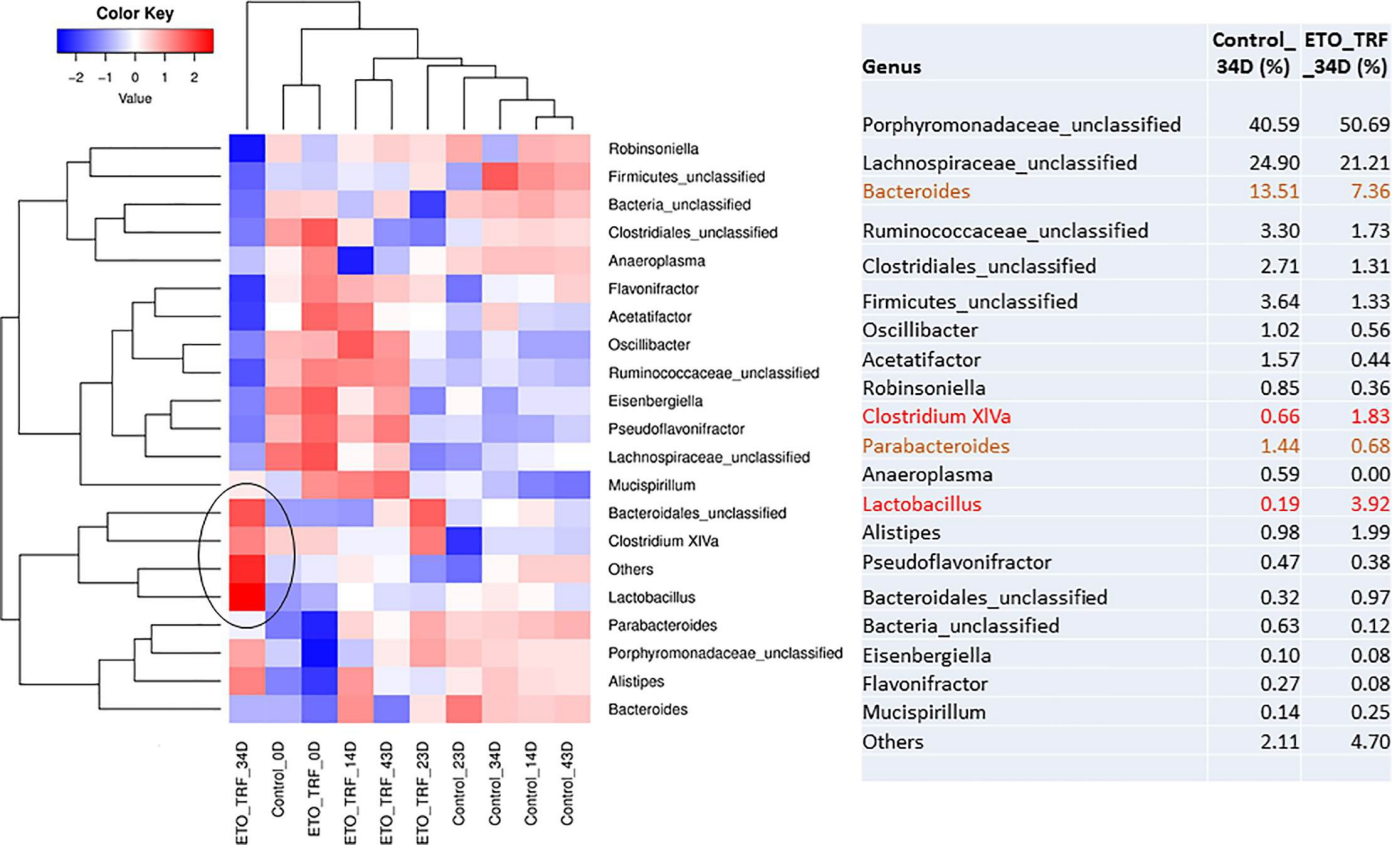
Heatmap showed the effect of ETO-Cur-TRF on relative abundance of microbial genus level in genomic DNA from mice feces. Red indicates high abundance and blue indicates less abundance. Marked increases in beneficial bacteria could be noted as indicated by red color after 34 days of ETO-Cur-TRF treatment. Black circle indicates increased abundance of specific microbial genus following ETO-Cur-TRF treatment.

16S gene profiling revealed that the abundance of *Bacteroides* and *Parabacteroides* was decreased in ETO-Cur-TRF treated mice while genus *Clostridium XIVa* was increased significantly (Fig 5). *Lactobacillus* also increased significantly with ETO-Cur-TRF as well as *Alistipes*. At species level, *Bacteroides Uniformis* were reduced in ETO-Cur-TRF treated mice.

**Fig 6:**
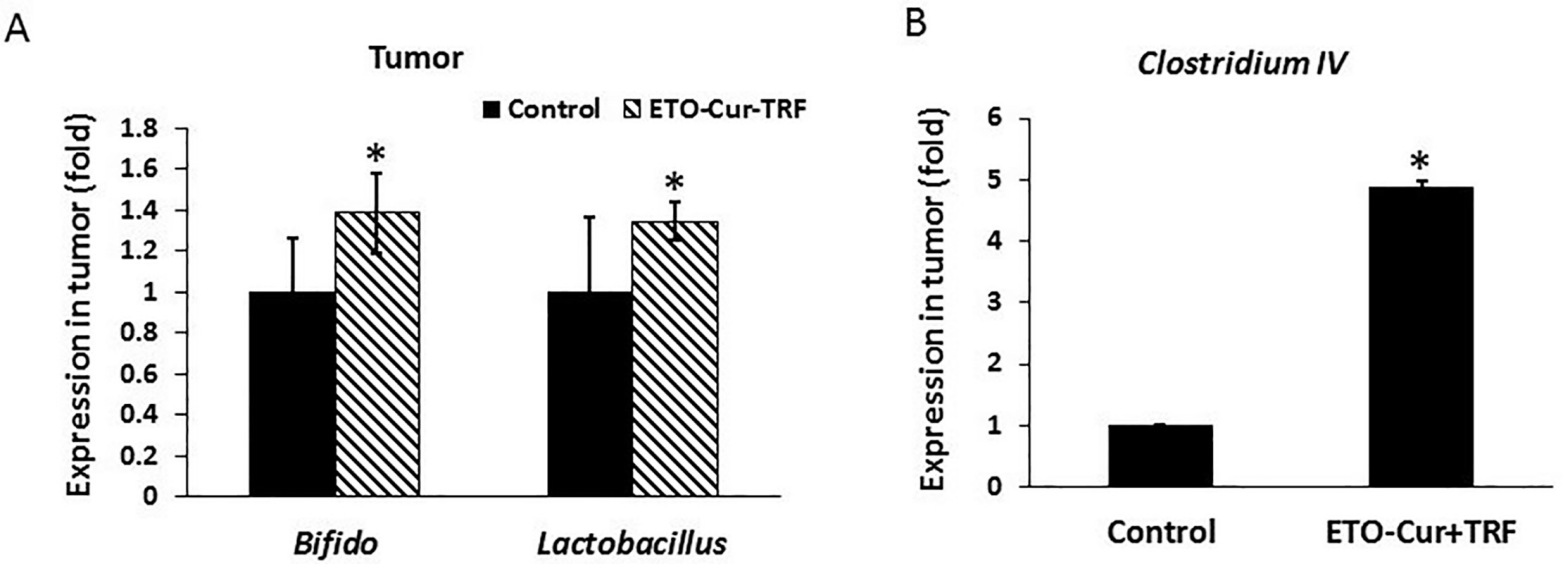
Changes in the relative abundance of (A) *Bifidobacteria* and *Lactobacillus* and (B) *Clostridium IV* in the xenograft of SCID mice 34 days following administration of Cur-ETO-TRF or vehicle (controls) as determined by qPCR (50 ng DNA/sample) with specific primers and each sample ^ΔΔ^CT values were calculated by normalizing to the CT value of total Eubacteria. Bars represent mean of three reading standard deviation; *p<0.01, compared to the corresponding control.

To determine whether gut microbial changes are reflected in the tumor, relative abundance of a limited number of bacteria in the mouse xenograft was performed by qPCR. Genomic DNA extracted from the tumors of SCID mice that were treated with ETO-Cur-TRF or vehicle (controls) for 34 days. DNA was assayed for *Bifidobacteriaceae* and *Lactobacilaceae* by qPCR with specific primers. Abundance of probiotic bacteria, *Bifidobateria*, *and Lactobacillus* in the tumor was found to be significantly higher in the ETO-Cur-TRF treated mice than their control (Fig 6A). Likewise, the relative abundance of beneficial bacteria *Clostridium IV* was also increased, compared to the control (Fig 6B). *Clostridium IV* is known to possess anti-inflammatory effects.[45–47]

## Discussion

While vitamin E isomers and Curcumin exhibit poor bioavailability, ETO-Cur, which is found to be 7–8 times more bioavailable than standard Curcumin synergized with TRF and together they greatly inhibited the growth of colon cancer cells *in vitro* and *in vivo*. It has been reported that the anti-inflammatory property of ETO-Cur is superior than standard Curcumin as evidenced by the improved disease activity index in dextran sodium sulfate–induced ulcerative colitis in mice.[15] Earlier studies also demonstrated that treatment with Curcumin or dietary polyphenols causes alterations in microbial composition accompanied by induction of mucosal immune cells.[6, 43, 48, 49] Our current data also demonstrate that administration of ETO-Cur-TRF modulates fecal microbial composition and increases microbial OTU biodiversity.

Analysis of 16S rRNA genes in cells isolated from feces from control and ETO-Cur-TRF treated mice revealed the phylogenetic structure of active microbial communities. Our current data show that ETO-Cur-TRF mediated alterations in microbial composition exhibited an increased abundance of *Porphymonadaceae*, *Firmicutes*, *Rickenellaceae*, *Lactobacillaeceae* and *Bifidobacteriaceae*; while *Bacteroidaceae*, *Lachnospirace* and *Ruminococcaceae* were decreased. These changes in bacterial populations in mice following ETO-Cur-TRF treatment clearly show that the current combinatorial treatment exerts a profound effect on the gut microbiome and suggest that the changes in microbial diversity are linked to changes in CRC proliferation. Future work will examine whether they have a role in inhibiting the growth of colon cancer cells in vitro and in vivo.

It has been reported that enrichment of *Bacteriodes* and reduction of members of the *Porphydomonaceae* family are associated with tumor growth in mice.[50] Our current observation that administration of ETO-Cur-TRF decreased *Bacteroides by about 45%* suggests that inhibition of tumor growth in response to the current combinatorial treatment could partly be attributed to reduction in *Bacteroides*. It has also been reported that *Bacteroides*, *Parabacteroides* and *Alistipes* are strongly associated with tumor growth, whereas several members of *Clostridium* cluster including *XIVa* are associated with reduction in CRC tumor.[44] In the current investigation, we also found a similar trend in that ETO-Cur-TRF induced inhibition of tumor growth was associated with a greater abundance of *Clostridium XIVa*.

The gut microbiome plays a pivotal role in maintaining epithelial health and immune homeostasis.[34] Certain bacterial populations serve a protective role and are considered to be important mediators of immune health. Consequently, loss of them can induce inflammation and tumorigenesis. Our study shows that the microbial community in the gut changes in response to ETO-Cur and TRF treatment. In addition, we show that the combination of ETO-Cur and TRF leads inhibits proliferation of colorectal cancer cells in vitro and in mouse xenografts in vivo.
